# Spruce Bark-Extracted Lignin and Tannin-Based Bioresin-Adhesives: Effect of Curing Temperatures on the Thermal Properties of the Resins

**DOI:** 10.3390/molecules26123523

**Published:** 2021-06-09

**Authors:** Sunanda Sain, Leonidas Matsakas, Ulrika Rova, Paul Christakopoulos, Tommy Öman, Mikael Skrifvars

**Affiliations:** 1 Swedish Centre for Resource Recovery, University of Borås, SE-501 90 Borås, Sweden; SSain@lincoln.ac.uk; 2 Biochemical Process Engineering, Division of Chemical Engineering, Department of Civil, Environmental and Natural Resources Engineering, Luleå University of Technology, SE-971 87 Luleå, Sweden; leonidas.matsakas@ltu.se (L.M.); ulrika.rova@ltu.se (U.R.); paul.christakopoulos@ltu.se (P.C.); 3 Materials and Production, RISE Research Institutes of Sweden, SE-943 33 Öjebyn, Sweden; tommy.oman@ri.se

**Keywords:** bioresin adhesive, lignin, wood panel, cross-linking, curing, thermogravimetric analysis

## Abstract

In this study, formaldehyde-free bioresin adhesives were synthesised from lignin and tannin, which were obtained from softwood bark. The extraction was done via organosolv treatment and hot water extraction, respectively. A non-volatile, non-toxic aldehyde, glyoxal, was used as a substitute for formaldehyde in order to modify the chemical structure of both the lignin and tannin. The glyoxal modification reaction was confirmed by ATR–FTIR spectroscopy. Three different resin formulations were prepared using modified lignin along with the modified tannin. The thermal properties of the modified lignin, tannin, and the bioresins were assessed by DSC and TGA. When the bioresins were cured at a high temperature (200 °C) by compression moulding, they exhibited higher thermal stability as well as an enhanced degree of cross-linking compared to the low temperature-cured bioresins. The thermal properties of the resins were strongly affected by the compositions of the resins as well as the curing temperatures.

## 1. Introduction

Recent environmental concerns have made researchers focus on the development of biobased materials in order to develop new valuable products and chemicals of natural origin. In the wood adhesive industry, synthetic resin adhesives such as phenol–formaldehyde (PF), urea–formaldehyde (UF), and melamine urea–formaldehyde (MUF) have dominated the market due to their easy processing, good mechanical performances, and low price [1]. These resin adhesives have major uses in wood panel production with plywood, particle boards, and medium-density fibreboards (MDF), which are very promising materials for the furniture, packaging, and construction sectors. However, these resins have formaldehyde as a major component, which is toxic and a potentially carcinogenic compound [2]. In PF resins, the phenol part is also an environmental hazard due to its toxicity, and the replacement of phenol has become a target for the industry. So far, many research works have been carried out in order to synthesise formaldehyde-free bioresin adhesives from biobased resources such as cellulose, lignin, and tannin [3].

After cellulose, lignin is the second most abundant renewable and naturally occurring biopolymer. It is a highly branched polymer consisting of phenolic monomer units and it can have an abundancy of up to 20–30% in lignocellulosic biomass, depending on its source [4]. The phenolic units in lignin are coniferyl alcohol, sinapyl alcohol, and cumaryl alcohol, which make lignin a potential precursor for the synthesis of bioresin adhesives [5]. Previous studies have shown that lignin could replace phenol in PF resin due to their structural similarity. The use of lignin could help to improve the physicochemical and thermal properties of PF resin as well as wood-based panels [6,7].

Currently, 50 million tons of lignin are produced per year from the pulping industry as waste (such as kraft lignin) [8]. The lignin side stream is normally incinerated to produce energy for the pulping mill, but there is an evident potential to use lignin as a raw material for chemicals and resins [9]. Apart from this type of lignin, the use of biorefinery technologies for lignocellulose processing result in the production of additional lignin as a by-product (such as hydrolysis lignin). More importantly, the emerging organosolv fractionation technologies can deliver lignin fractions that are of high purity, practically ash-free, and chemically closer to native lignin [10]. These properties allow for better valorisation of lignin towards high-added value products. Thus, it is apparent that the use of lignin to develop new value-added materials is a promising approach for supporting an economically beneficial circular economy. However, the complex and branched polymeric structure of lignin has led to its poor chemical reactivity in resin synthesis. This could be overcome via different chemical modifications of its structure [11]. Most of these chemical modification reactions involve demethylolation, phenolation, and methylolation steps, which could generate reactive sites into lignin structure and transform lignin into various phenolic compounds [9]. This chemically modified lignin structure could undergo oxidation and polymerisation reactions easily, and as a result the modified lignin could be suitable to use as wood adhesives [12]. These chemical modification reactions (particularly methylolation) involve formaldehyde, which should be avoided in any new bioresin intended to be environmentally friendly and sustainable [11]. A substitute for formaldehyde is the dialdehyde glyoxal, which is an environmentally friendly option for the chemical modification of lignin. It is a natural resource-based compound, obtained as a by-product from biological processes and lipid oxidation [13]. Glyoxal has a low vapour pressure and low toxicity, and its two adjacent carbonyl groups make it reactive. Glyoxal undergoes similar reactions as formaldehyde with phenolic rings and has therefore been used as formaldehyde replacement in wood adhesives. The glyoxalation of lignin and the curing of tannin with glyoxal have already been introduced as an effective method for resin adhesive synthesis [14].

**Figure 1:**
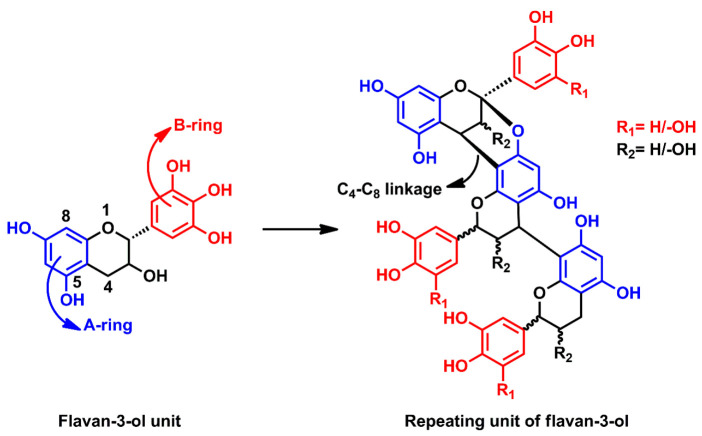
Structure of proanthocyanidine or condensed tannin [16].

Tannin resins have been widely used in the wood industry since the 1970s [15]. Tannin is a polyphenolic compound, extracted from the bark, roots, fruits, and leaves of several plants. Softwood bark usually contains condensed tannins or proanthocyanidines [16]. Proanthocyanidines are the repeating units of flavan-3-ol (Figure 1), consisting of A and B type rings which are responsible for the adhesive and antioxidant characteristics of tannins, respectively. Furthermore, tannin has extensive potential to substitute phenol in PF resins due to the presence of multiple polyhydroxyl–phenyl groups in its structure. Previous studies have revealed that condensed tannins show higher reactivity towards formaldehyde compared to phenol. Konai et al. have formulated resin adhesives using tannin and various hardeners like paraformaldehyde, glyoxal, and hexamine [14]. Different resin adhesives have been formulated using glyoxalated lignin in combination with tannin to develop fully biobased resin adhesives [17]. The crude tannin, which has not been further purified, contains sugars and gums. These could affect the final strength and water resistance property of the resins [15]. To the best of our knowledge, most reported works have utilised crude tannin instead of purified tannin because purification by SPE (solid phase extraction) is a costly and cumbersome process due to the difficulties involved in choosing the right solvent and obtaining concentrated samples after purification [18]. In this respect, the use of chemically modified tannins would be a wise choice over crude tannin in order to overcome the issues with the purification of tannin and to minimise the effect of gums and sugars at the same time [15].

Significant amounts of bark are potentially available from the forest industry in Europe as by-products of pulp and sawmills and as forest harvest residues from logging operations. Although this bark is mainly used for fuel or soil improvement in gardening, it could have a potential for the production of biochemicals. One of the main goals of this study is to develop a holistic method for the use of non-carbohydrate components of bark in order to produce bio-based environmentally friendly adhesives, taking advantage of the significant amounts of bark available from the forest industry in Europe. The tannin and lignin content of spruce bark is approximately 10.7% and 26.8%, thereby offering a valuable source for lignin and tannin valorisation. After a cascade type tannin and lignin extraction in several steps, we used chemically modified tannin along with chemically modified lignin for the bioresin adhesive synthesis. The main objective of this work was to synthesise formaldehyde-free and water-resistant bioresin from spruce bark-derived lignin and tannin. Three different combinations of these chemically modified raw materials (lignin and tannin) were examined for the resin synthesis. The thermal properties of these resins have been evaluated and compared in detail in order to find out the best composition for the bioresin adhesive.

## 2. Results and Discussion

Noncarbohydrate components (lignin and tannin) of softwood bark were utilised to formulate different bioresin adhesives, avoiding the use of toxic formaldehyde. The chemical functionality for lignin and tannin allows a similar curing reaction with glyoxal as for formaldehyde. Consequently, the extracted lignin (L), tannin (T), and lignin–tannin mixtures (LT) were chemically modified with a non-toxic dialdehyde, glyoxal (GO). Different bioresin adhesives were then formulated with chemically modified L, T, and LT (i.e., GOL, GOT, and GOLT) and hexamine (H), which served as a curing agent.

**Figure 2:**
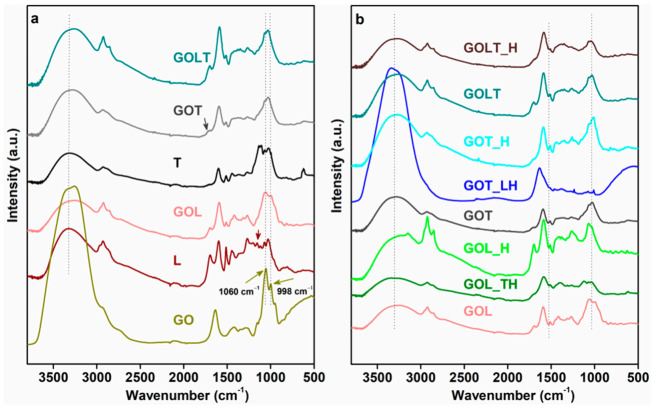
FTIR plots of (**a**) L, T, and their glyoxalated forms (The green arrows indicate the peaks from glyoxal at 1060 and 998 cm^−1^, while the grey arrow in GOT at 1718 cm^−1^ indicates carbonyl stretching from glyoxal moiety) and (**b**) cured resins.

**Figure 3:**
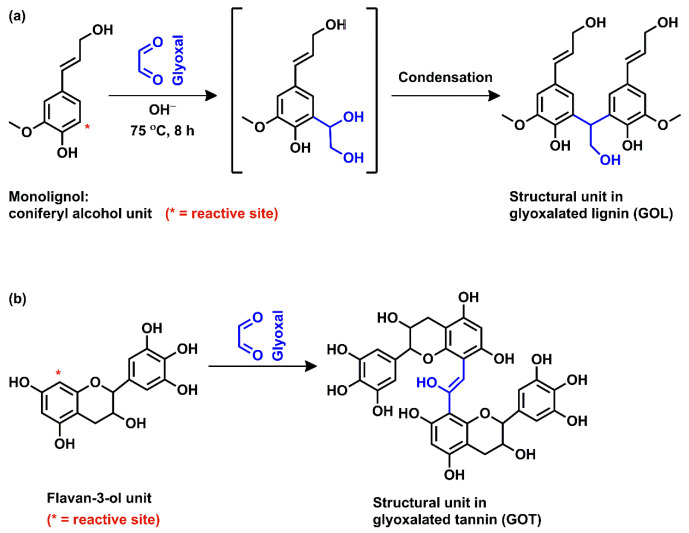
Probable reaction schemes between (**a**) glyoxal and lignin [22] and (**b**) glyoxal and tannin [23].

The chemical reaction of glyoxal moiety into the raw materials was confirmed by FTIR analysis. Figure 2a compares the FTIR spectra of L, T, GOL, GOT, and GOLT. The presence of a broad signal at 3320 cm^−1^ attributed to the –OH stretching due to phenolic structure in L, T, and modified components (GOL, GOT, and GOLT) [11]. After glyoxal modification of the components, the –OH stretching shifted to 3262 cm^−1^ in GOL, GOT, and GOLT. This might be due to the interaction between the glyoxal group and phenolic –OH group. Presence of glyoxal peaks at 998 cm^−1^ and 1060 cm^−1^ in GOL, GOT, and GOLT confirmed the incorporation of glyoxal moiety into the L, T, and LT mixtures [19]. An increase in the intensity of these peaks suggested that the reaction happened through the glyoxal carbonyl group and phenolic –C–H group in lignin, as expected. The small characteristic peak at 1157 cm^−1^ in lignin disappeared after the glyoxalation reaction, which indicates a cleavage of β–O–4 and α–O–4 linkage due to demethoxylation. In GOT, a small hump was observed at 1718 cm^−1^, which was due to >C=O group from glyoxal [20]. The signal for –C–O stretching and –C–C bending of the aromatic structure at 1140 cm^−1^ disappeared at GOT [20]. The intensity of aromatic skeletal vibration at 1512 cm^−1^ decreased in GOL, GOT, and GOLT after the glyoxalation reaction [21]. The probable reaction schemes for glyoxalation of lignin and tannin are shown in Figure 3.

**Figure 4:**
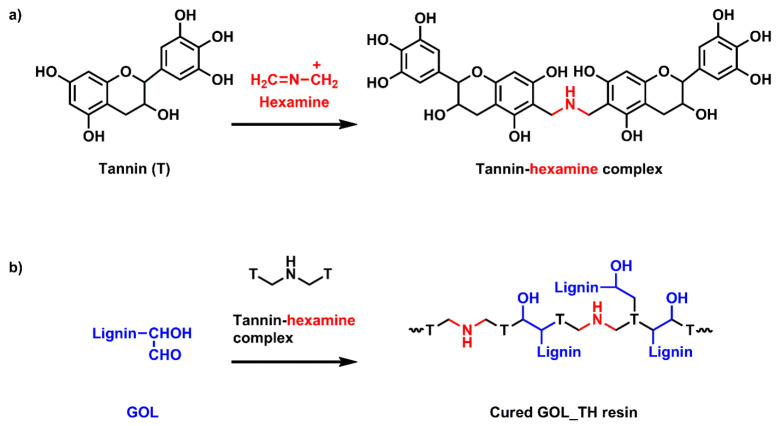
Probable reaction schemes for curing of GOL_TH resins: reaction between (**a**) tannin and hexamine [1] and (**b**) GOL and tannin–hexamine complex [25].

In the cured resin (Figure 2b) GOL_TH, the spectrum was similar to the spectrum for GOL. The only significant difference was in their peak intensities. The reduced intensities of the characteristic peaks suggest a curing reaction between glyoxalated lignin and tannin–hexamine system. In GOL_H, the broad stretching at 3302 cm^−1^ was shifted to a lower wavenumber region at 3219 cm^−1^, which might be due to the hydrogen bonding between –NH from hexamine and –OH from GOL [24]. Other signals in GOL_H were almost the same as with GOL. The enhanced intensity of the –OH stretching signal at 3312 cm^−1^ in GOT_LH attributed to the increase in the phenolic –OH group, due to the incorporation of lignin. Limited signals at the 1000–1500 cm^−1^ region in the GOT_LH sample suggest either the delocalisation of π electrons or the formation of polymerised structure due to curing of the resin [20]. The spectral signals were almost the same for GOLT and GOLT_H. The broadening of the signals in 1400–1500 cm^−1^ region in GOLT_H might be due to the crosslinking reaction between hexamine and glyoxalated LT. The disappearance of the 1505 cm^−1^ peak from GOT_LH and GOLT_H, and the very low intensity signal at this region for GOL_TH, suggest the formation of a highly cross-linked structure. A probable crosslinking reaction between GOL and tannin–hexamine has been shown in Figure 4.

**Figure 5:**
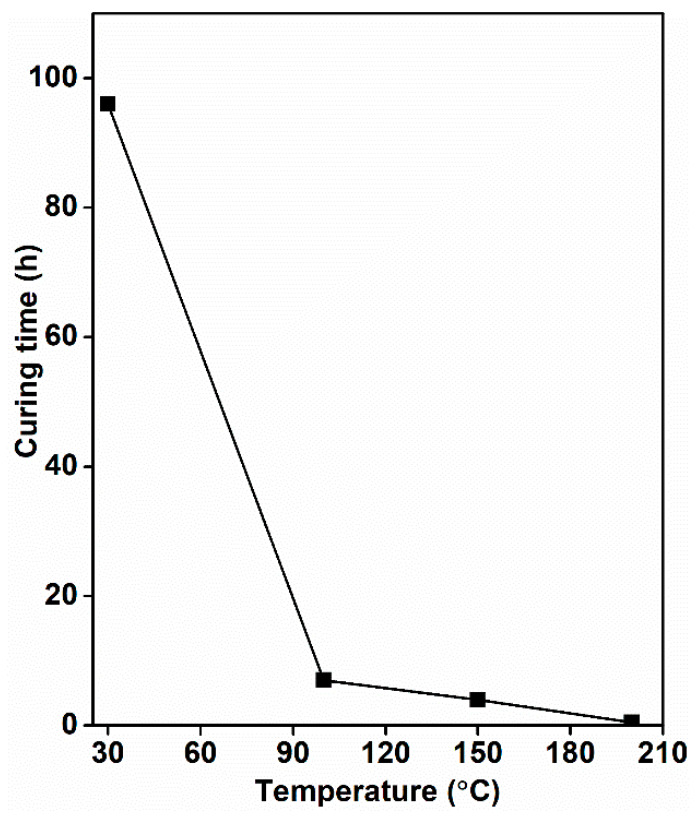
Variation of curing time with temperature for Gol_TH resins.

The effect of temperatures on the curing of the resins was examined and, therefore, GOL_TH resins were cured at different temperatures (30, 100, 150, and 200 °C) in order to find out the best curing temperature for these bioresins. It was observed that low temperature (30 °C) curing of GOL_TH resin took four days (96 h), while high temperature curing (200 °C) of these resins reduced the curing time to 30 min (Figure 5).

**Figure 6:**
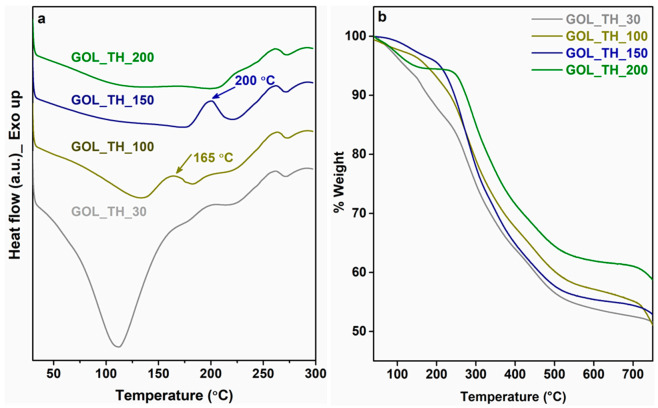
(**a**) DSC plots of GOL_TH resins cured at different temperatures (first heating scans; the blue and green arrows indicate the exotherms at 200 °C and 165 °C, respectively) and (**b**) TGA plots of GOL_TH resins cured at different temperatures.

Curing of the resins was studied through DSC, and the thermal stability of the cured resins was assessed by TGA. According to Figure 6a, the GOL_TH resin cured at 30 °C showed a sharp endotherm at 111 °C, which could be attributed to the evaporation of moisture as well as any unreacted components still present. On the other hand, the exothermic transitions at 165 °C and 200 °C for the cured resins GOL_TH_100 and GOL_TH_150, respectively, suggest that the resins were not cured completely and that some functional groups were still free to undergo further chemical reaction. The absence of any exothermic or endothermic transition for the GOL_TH resin cured at 200 °C suggest the complete curing of the resins with thermally stable crosslinked networks, which was further confirmed via TGA analysis. From the TGA results (Figure 6b), it was observed that the GOL_TH resin cured at 200 °C exhibited the formation of an enhanced crosslinked structure with a ~60% char formation and a higher thermal stability than other GOL_TH resins cured at 30, 100, and 150 °C. Furthermore, the reduced curing time at this 200 °C was an added advantage. Based on these results, therefore, a temperature of 200 °C was considered as the suitable curing temperature and the rest of the resins were cured at 200 °C.

**Figure 7:**
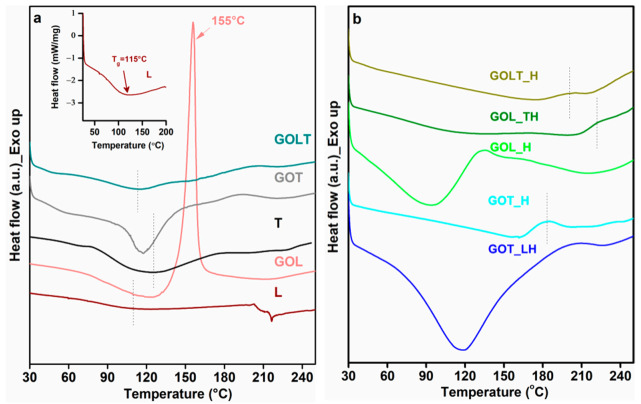
DSC plots (first heating scans) of (**a**) lignin, tannin, GOL, GOT, and GOLT and (**b**) cured GOL, GOT, and GOLT resins.

In Figure 7a, the DSC plots showed a small transition around 110 °C for lignin, which could be attributed as the glass transition temperature (T_g_) of the bark-derived lignin. However, the endothermic glass transition at ~95 °C in GOL was likely masked by the sharp exothermic transition at 159 °C. A decrease in the molecular weight and polydispersity in the lignin molecules after glyoxal modification [7], and an increase in the free volume of the molecules due to the incorporation of aldehyde substituent into the system, resulted in a reduced T_g_. The incorporation of aldehyde substituent in the lignin structure enhanced the chance of self-condensation reaction between the components in the alkaline medium [7]. A sharp exothermic transition at 159 °C indicated that functional groups or reaction sites were still available in GOL for further reaction due to the introduction of more hydroxyl groups (–CH_2_OH) via glyoxal modification [26]. Determination of T_g_ in the case of the pure dried tannin sample was difficult due to its complex structure. Bark-derived pure tannin showed a wide endothermic transition at 126 °C, which was probably due to the glass transition region. After the glyoxal modification of tannin (GOT), this T_g_ was shifted to 116 °C. This could be explained in a similar way as GOL. A small exothermic hump at 193 °C suggests that there were free reacting sites which were available for a curing reaction with hexamine. After glyoxal modification of the lignin–tannin mixture (GOLT), the T_g_ was observed at 113 °C, which is close to the T_g_ of GOT. Therefore, it can be concluded that tannin is more reactive towards glyoxalation reaction than lignin.

In Figure 7b, small exothermic humps were observed in the DSC scans for GOT_H, GOLT_H, and GOL_TH at 183 °C, 200 °C, and 220 °C, respectively, which indicates a curing reaction between the components. After curing, there were still free amine groups left for further reaction. In GOT_LH and GOL_H, the absence of any exothermic transition confirms the complete curing of the resins. The broad endothermic transitions, at 94 °C for GOL_H and at 117 °C for GOT_LH, might be due to the evaporation of water generated via condensation reactions between amine groups and hydroxyl groups.

**Figure 8:**
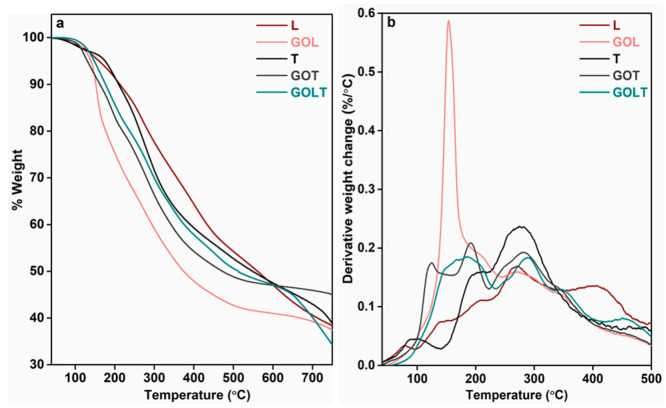
(**a**) TGA and (**b**) DTG plots of lignin, tannin, GOL, GOT, and GOLT.

Figure 8a,b shows the TGA and DTG curves of the lignin and tannin samples. There were no sharp degradation peaks in the bark-derived lignin, tannin, glyoxalated tannin (GOT), and glyoxalated lignin–tannin (GOLT) samples. Instead, only small humps were observed. According to the DTG curves (Figure 8b), the pure lignin showed five small degradation humps at 80 °C, 141 °C, 208 °C, 269 °C, 405 °C, and 609 °C, respectively, whereas the glyoxalated lignin (GOL) showed a sharp degradation peak at 153 °C and three small humps at 270 °C, 344 °C, and 470 °C. The sharp degradation peak at 153 °C might correspond to the degradation of short chain lignin molecule generated via condensation reaction during the glyoxalation of lignin [27]. The tannin showed three stages of degradation at 95 °C, 198 °C, and 276 °C, whereas the GOT showed humps at 123 °C, 191 °C, 282 °C, and 350 °C. For the GOLT, a broad peak was observed at 175 °C, and three small humps were observed at 290 °C, 353 °C, and 460 °C. The presence of multi shoulders in the DTG curves indicate the degradation steps of different structural units present in the lignin [28]. A small hump below 100 °C in both the lignin and tannin indicates the evaporation of moisture. In the GOL, GOT, and GOLT samples, from 100–200 °C the degradation occurred due to breaking of side chains and small carbohydrate molecules. Above 300 °C, the weight loss occurred due to a loss of moisture, generated by a condensation reaction between the methylene group and phenolic –OH [29]. After modification, the thermal stability of the GOL, GOT, and GOLT samples decreased more than both pure lignin and tannin.

**Figure 9:**
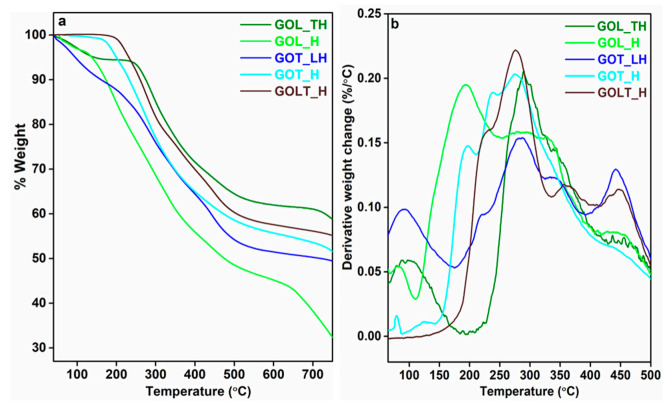
(**a**) TGA plots of cured resins and (**b**) DTG plots of cured resins.

From Figure 9a, it can be observed that from room temperature to 200 °C the thermal stability is initially lower in GOL_TH and GOT_LH than the other cured resins. This might be due to the evaporation of volatiles, generated due to chemical reaction between hexamine and other components in the system. The GOLT_H resin showed degradation peaks at 225 °C, 276 °C, 362 °C, and 448 °C (Figure 9b). The presence of phenolic functionalities from lignin, tannin, GOL, and GOT helped in the formation of methylene bridges with hexamine by developing cross-linked polymeric structures [15]. The formation of cross-linked structures is also reflected in the number of residues mentioned in Table 1. In the case of GOT_H and GOL_H, small degradation peaks were observed at 196 °C, 238 °C and 276 °C, and 84 °C, 194 °C, and 448 °C, respectively (Figure 9b), which could be due to the degradation of side chains of the resins and the formation of volatile components from reaction with hexamine [14]. Thus, it was observed from the results that the resin containing both lignin and tannin functionalities will give the best cross-linking properties [15].

## 3. Experimental Section

### 3.1. Materials

Spruce bark was provided by Sveaskog and used as a source of lignin and tannin. Bark was air-dried and milled through a 1 mm screen in a knife mill (Retsch SM 300, Retsch GmbH, Haan, Germany) and stored in sealed bags at room temperature until further use. The moisture content of the milled bark was 7.9% *w*/*w* [30]. Sodium hydroxide (NaOH), glyoxal solution (GO, 40 wt% solution in water), and hexamethylene tetramine (ACS reagent > 99.0%) were used in the synthesis. All of these chemicals were purchased from Sigma Aldrich (St. Louis, MO, USA).

### 3.2. Tannin and Lignin Extraction from Softwood Bark

The milled bark was subjected to hot water extraction in order to extract tannin in a multi-digester system that contains six 2.5 L metallic cylinders and was used as previously described [30]. Specifically, 110 g of bark was mixed with 1.1 L of water in which 2% *w/w*_solids_ sodium bisulphite and 0.5% *w/w*_solids_ sodium carbonate were added to facilitate the tannin extraction. Treatment took place for 2 h at 75 °C. At the end of the treatment, the tannin-extracted bark solids were separated by vacuum filtration from the liquor containing the extractives, washed with distilled water, and air-dried until further use. The processed liquor was evaporated in a rotary evaporator in order to remove the water. Finally, it was freeze-dried to yield a tannin-rich powder (consisting of 30.2% *w*/*w* of the initial bark) [30]. The tannin-rich powder contained 48.1% *w*/*w* of tannins [30], analysed according to the protocol suggested elsewhere, with the difference in phenolic compounds determined by using the Folin–Ciocalteu reagent instead of the Folin–Denis reagent [31,32]. To produce lignin, tannin-extracted bark was subjected to organosolv fractionation in the same reactor where the hot water extraction took place. More specifically, 90 g of tannin-extracted bark was mixed with 900 mL of 60% *v*/*v* ethanol in a water solution and treated for 1 h at 180 °C. At the end of the treatment, the slurry was filtrated under a vacuum in order to separate the pretreated solids with the liquor. The liquor was collected and ethanol was removed in a rotary evaporator, followed by centrifugation in order to recover lignin. Lignin was then air-dried and stored at room temperature until further use. The contamination of lignin, determined according to the NREL technical report [33], was (%, *w*/*w*): cellulose, 1.5; hemicellulose, 0.6; and ash, 0.2 [34].

### 3.3. Chemical Modifications

Extracted lignin and tannin were chemically modified with glyoxal.

*(a) Glyoxalation of spruce bark derived lignin.* The glyoxalation of lignin in an alkali medium was carried out in a three-necked round bottom flask fitted with a reflux condenser and a thermometer [11]. Lignin was mixed with water at a ratio of 1:2 (*w*/*w*), and the pH of the mixture was adjusted to 12.5 by 30% (*w*/*v*) of NaOH solution. The mixture was heated at 65 °C and incubated under continuous stirring for 30 min. Then, glyoxal solution (60% *w/w*_lignin_) was added and the final reaction mixture was heated at 75 °C and maintained at that temperature for 8 h. At the end of the reaction, the product was cooled down and filtered in order to obtain glyoxalated lignin (GOL).

*(b) Glyoxalation of spruce bark derived tannin*. The tannin extracted from spruce bark was modified with glyoxal in the same procedure described in section (a). The amount of glyoxal used here was 25% *w/w*_tannin_. Thus, glyoxalated tannin (GOT) was obtained.

*(c) Glyoxalation of spruce bark derived lignin–tannin mixture*. Glyoxal modification of the lignin–tannin mixture was performed following the same reaction conditions described in section (a). The lignin:tannin ratio in the mixture was 2:3 (*w*/*w*). Glyoxalated (lignin–tannin) mixture (GOLT) was obtained in this reaction.

### 3.4. Resin Adhesive Formulation

Resin adhesives were formulated using GOL, GOT, and GOLT. For the GOL set, the ratio of GOL:tannin in the mixture was 2:3 (*w*/*w*). More specifically, a 45% (*w*/*v*) tannin solution was added to GOL, and the pH of the medium was adjusted to 10 [29]. After that, 33% (*w*/*v*) solution of a curing agent, hexamine (H) 6% (*w*/*v*), was added in the GOL–tannin mixture. The resins were cured in an oven using mini aluminium muffin cups. The oven was set at 30 °C, 100 °C, 150 °C, and 200 °C, and corresponding curing times were recorded. The curing time determination tests were performed in triplicates. While similar curing procedures were followed for GOT and GOLT sets, these resins were cured only at 200 °C. This was considered as the best curing temperature because of the reduced curing time. All sample details and reaction conditions have been described in Table 2.

The gelation of the GOL_TH_100 resin was carried out at by a shaking water bath with a set temperature at 100 °C. A stopwatch was used to record the gel time. The gel time of GOL_TH resin at 100 °C was 15 min.

### 3.5. Characterisation

Chemical modification reactions of the lignin, tannin and lignin–tannin mixtures were confirmed by FTIR spectroscopy. An attenuated total reflectance Fourier transform infra-red spectrometer (ATR–FTIR), Nicolet is 10, Thermofisher Scientific (Waltham, MA, USA), was used. Differential scanning calorimetry (DSC Q1000, TA instruments, Newcastle, UK) was used to study the curing behaviour of the modified lignin, tannin, and lignin–tannin mixtures and formulated resins. Tzero aluminium pans were used in DSC and the samples were run at standard DSC mode in open pans. The experiments were carried out under a nitrogen atmosphere at a temperature range of 25–250 °C with a 10 °C/min heating rate. Data from the first heating scan were recorded. The thermal stability of the resins was studied by thermogravimetric analysis (TGA Q1000, TA instruments, Newcastle, UK) under a nitrogen atmosphere within a temperature range of 30–800 °C at a 10 °C/min heating rate.

## 4. Conclusions

In this study, we have demonstrated the full-fledged use of softwood bark-derived lignin and tannin (the non-carbohydrate components) to synthesise bioresin adhesives. The chemical structure of the extracted lignin and tannin were modified with glyoxal, a non-toxic aldehyde. Three different combinations of these glyoxal modified lignin and tannin (i.e., GOL, GOT, and GOLT) were examined in order to formulate suitable resin adhesives. The presence of characteristic FTIR signals of glyoxal in GOL, GOT, and GOLT confirmed the modification of lignin, tannin and the mixture of lignin–tannin with glyoxal. Curing of these resins and the degree of cross-linking were strongly influenced by the temperature. Higher temperature curing ensured the higher cross-linking between the resin components. Thus, the GOL_TH resin cured at 200 °C showed a lower curing time (30 min) along with an enhanced degree of cross-linking and thermal stability. Resin composition also had a strong influence in the variation of both the thermal and mechanical properties of the resins. The modification of the lignin structure strongly influenced the resin curing reaction of the resins. Thus, it is possible to formulate sustainable, water resistant, and thermally stable cross-linked resin adhesives from spruce bark-derived lignin and tannin upon modification with glyoxal, which has the potential to replace formaldehyde.

## Figures and Tables

**Table 1:** TGA results of samples.

Samples	T_10_ (°C)	T_30_ (°C)	T_50_ (°C)	% Residue
L	211	358	561	39
T	209	308	545	45
GOL	150	231	374	37
GOT	164	279	471	38
GOLT	177	298	509	34
GOL_TH	276	416	-	58
GOL_H	172	291	472	28
GOT_LH	160	344	711	48
GOT_H	232	346	762	46
GOLT_H	259	397	800	51

T10, T30, and T50 denote the degradation temperatures corresponding to 10%, 30%, and 50% weight loss, respectively.

**Table 2:** Resins and curing conditions of resins.

Sample Names	Compositions (wt%)	Hexamine (H) (wt%)	Curing Temperature (°C)	Curing Time (h)
GOL_TH_30	GOL (40%), T (60%)	6	30	96
GOL_TH_100	GOL (40%), T (60%)	6	100	7
GOL_TH_150	GOL (40%), T (60%)	6	150	4
GOL_TH_200	GOL (40%), T (60%)	6	200	0.5
GOL_H_200	GOL	6	200	0.5
GOT_LH_200	GOT (40%), L (60%)	6	200	0.5
GOT_H_200	GOT	6	200	0.5
GOLT_H_200	GOLT	6	200	0.5

## Data Availability

Data are available upon request to corresponding author.

## Sample Availability

Lignin, tannin, glyoxal modified lignin and tannin samples are available from the authors.
